# Genetics of vestibular disorders: pathophysiological insights

**DOI:** 10.1007/s00415-015-7988-9

**Published:** 2016-04-15

**Authors:** Lidia Frejo, Ina Giegling, Roberto Teggi, Jose A. Lopez-Escamez, Dan Rujescu

**Affiliations:** Otology and Neurotology Group CTS495, Department of Genomic Medicine, GENYO - Centre for Genomics and Oncological Research – Pfizer/University of Granada/Junta de Andalucía, PTS, 18016 Granada, Spain; German Center for Vertigo and Balance Disorders, Munich, Germany; Department of Otolaryngology, San Raffaelle Scientific Institute, Milan, Italy; Department of Otolaryngology, Granada University Hospital, 18012 Granada, Spain

**Keywords:** Vestibular disorders, Whole exome sequencing, Vestibular migraine, Motion sickness, Meniere disease

## Abstract

The two most common vestibular disorders are motion sickness and vestibular migraine, affecting 30 and 1–2 % of the population respectively. Both are related to migraine and show a familial trend. Bilateral vestibular hypofunction is a rare condition, and some of patients also present cerebellar ataxia and neuropathy. We present recent advances in the genetics of vestibular disorders with familial aggregation. The clinical heterogeneity observed in different relatives of the same families suggests a variable penetrance and the interaction of several genes in each family. Some Mendelian sensorineural hearing loss also exhibits vestibular dysfunction, including DFNA9, DFNA11, DFNA15 and DFNA28. However, the most relevant finding during the past years is the familial clustering observed in Meniere’s disease. By using whole exome sequencing and combining bioinformatics tools, novel variants in *DTNA* and *FAM136A* genes have been identified in familial Meniere’s disease, and this genomic strategy will facilitate the discovery of the genetic basis of familial vestibular disorders.

## Introduction

Vestibular disorders (VDs) are considered a group of diseases leading to transient or permanent loss of vestibular function. Regardless of their clinical heterogeneity, several families with vestibular symptoms affecting different relatives have been described. However, the contribution of genetic variations, either common or rare variants, to develop a specific vestibular disease is largely unknown. Some of the reasons for this missing heritability in VDs are the limitations in clinical phenotyping, the overlapping symptoms among different VDs and the clinical heterogeneity observed in families with variable penetrance (Table 1). According to their clinical presentation, we can distinguish three major syndromes: an episodic vestibular syndrome, a progressive vestibular syndrome and a third group of diseases, which include sensorineural hearing loss (SNHL) with a variable vestibular dysfunction [1, 2].

**Table 1:** Factors leading to underdiagnosis of familial vestibular disorders

Poor clinical phenotyping
Lack of knowledge of the hereditary component in VDs by ENT/neurologists
Misdiagnosis of family members with VDs (vestibular migraine or Meniere’s disease)
Inadequate recording of pertinent family history of vertigo or hearing loss
Clinical variability
Clinical heterogeneity of VD in different families
Small family size
Incomplete disease penetrance (AD, mitochondrial inheritance), partial phenotype

In this review, we will update the evidences to support the genetic contribution in some of the vestibular disorders with familial aggregation.

## Episodic vestibular syndromes

The two most common vestibular disorders are motion sickness and vestibular migraine, affecting 30 and 1–2 % of the population, respectively. Both are complex, multifactorial disorders with a strong genetic contribution. Table 2 summarizes diseases with vestibular involvement and potential genes involved.

**Table 2:** Vestibular disorders and potential genes involved

Disorder	Gene or locus	References
Episodic vestibular syndromes		
Motion sickness	*PVRL3*, *GPD2*, *ACO1*, *AUTS2*, *GPR26*, *UBE2E2*, *CBLN4*, *BLOC1S5*, *LINGO2*, *CPNE4*	[4]
Vestibular migraine	*CACNA1A*, *ATP1A2*, *SCN1A*, *KCNB2*, *CACNB2*	[11–13, 16]
Progressive vestibular syndromes		
Bilateral vestibular hypofunction	A region in chromosome 6q	[20]
Cerebellar ataxia, neuronopathy and vestibular areflexia syndrome (CANVAS)	Unknown	
Sensorineural hearing loss with variable vestibular dysfunction		
DFNA9	*COCH*	[24–27]
DFNA11	*MYO7A*	[30–33]
DFNA15	*POU4F3*	[34]
DFNA28	*GRHL2*	[35]
DFNB102/103	*CLIC5*	[38, 39]
EVAS	*SLC26A4*	[40–46]
Ménière’s disease	*MICA*, *TLR10*, *NFKB1*, *AQP2*, *AQP4*, *AQP3*, *KCNE1*, *KCNE3*, *ADD1*	[53–55, 57, 59–62]
Familial Ménière’s disease	5q14–15, 12p12.3, *DTNA*, *FAM136A*	[64–66]

### Motion sickness

Motion sickness refers to autonomic signs and symptoms occurring during movement, and maybe elicited by self-motion or motion of the environment. It is the most common vestibular condition affecting 30 % of the population. Its symptoms include dizziness, nausea, vomiting, pallor and headache [3]. The condition has been associated with postoperative vomiting, altitude sickness, morning sickness and migraine. A genome-wide association study performed in 80494 individuals has identified 35 common single-nucleotide variants at genome-wide significant level. The top ten genes involved in these regions include *PVRL3*, *GPD2*, *ACO1*, *AUTS2*, *GPR26*, *UBE2E2*, *CBLN4*, *BLOC1S5*, *LINGO2* and *CPNE4*. These genes involve a large variety of functions such as ocular and brain development, insulin resistance, otolith biogenesis or iron homeostasis [4].

### Vestibular migraine

Definite vestibular migraine (VM) is defined by the occurrence of episodic vestibular symptoms and a history of migraine, demonstrating a temporal association between vestibular and migraine symptoms in at least 50 % of the attacks [5]. VM shows a transient vestibular dysfunction, and it presents overlapping symptoms with Meniere’s disease (MD) during the attacks [6]. Moreover, the association with migraine, episodic vertigo and MD, frequently observed clustered in families, including identical twins, supports the heritability of VM [7].

Epidemiological studies report that around 56 % of MD patients also refer migrainous headaches [8]; in both disorders, a familial predisposition has been postulated. Migraine, *per se*, may provoke episodic vertigo, and VM has a prevalence of about 1 % of the population [5].

So, genetic variations in ion channels and membrane transporters which regulate fluid homeostasis could explain the relationship between migraine and MD [9, 10].

Different mutations in three genes have been linked to a rare form of migraine called familial hemiplegic migraine (FHM): (a) *CACNA1A*, provoking a gain of function in a L-type Ca^++^ channel, (b) *ATP1A2*, leading to a loss of function of the Na^+^, K^+^, -ATPase transporter and (c) *SCN1A* causing a gain of function of a voltage-gated Na^+^ channel [11–13]. Based on the observation of families presenting both FHM and common migraine, a shared pathophysiology has been postulated. The cortical spreading depression, for example, has been associated with acute changes in brain extracellular ionic concentration [14]; moreover, an increased capillary endothelial cell Na^+^, K^+^, -ATPase activity, leading to an increased Na^+^ concentration, has been linked to migraine pathophysiology [15]. In a recent paper, common variations in different ion transport genes were examined; none were significant, but an epistatic interaction between *KCNB2* gene (encoding a potassium channel) and *CACNB2* (encoding a calcium channel) was observed [16]. Conversely, no evidence of an association with genes encoding ion channels linked to FHM has been found in VM [17].

Ionic homeostasis in the inner ear plays an important role for the maintenance of the endocochlear potential. In Jervell and Lange-Nielsen syndrome, a mutation of *KCNQ1* and *KCNE1* channels leads to a severe SNHL and a collapse of the cochlear scala media [18].

More recently, a variant of *ATP1A2* has been associated in a Korean family with a new form of progressive hearing loss with migraine [19].

## Progressive vestibular syndromes

### Bilateral vestibular hypofunction

Bilateral vestibular hypofunction starts in young adults with episodes of vertigo, triggered by exercise and stress, causing chronic disequilibrium, postural instability and disabling oscillopsia. Bilateral vestibular hypofunction without hearing loss, also referred as bilateral vestibulopathy, was described in 20 families with autosomal dominant inheritance without migraine. Linkage analyses in these families segregated a region in chromosome 6q [20]. No genes have been identified in bilateral vestibular hypofunction.

### Canvas

Cerebellar ataxia, neuropathy and bilateral vestibular areflexia syndrome (CANVAS) is a neurodegenerative ganglionopathy. It is a rare late-onset slowly progressive ataxia, characterized by the combination of imbalance due to cerebellar gait and limb ataxia, bilateral vestibular impairment, and nonlength-dependent sensory neuropathy [21]. It has been reported a loss of neurons from the dorsal root and cranial nerve ganglia, but there is a phenotypic heterogeneity in CANVAS patients [22]. Although most of cases are sporadic, few families with several cases suggest genetic heterogeneity. No causal gene has been involved with CANVAS.

## Sensorineural hearing loss with variable vestibular dysfunction

Dizziness and episodic vertigo can be combined with moderate or severe hearing loss as a result of a cochleovestibular disorder. So, genes involved in the development of the otic capsule and temporal bone development are likely to cause cochleovestibular disorders. Hereditary deafness may be conductive, sensorineural or a combination of both: syndromic (associated with malformations of the external ear or other organs or with medical problems involving other organ systems) or nonsyndromic (without association to visible abnormalities of the external ear or any related medical problems); and prelingual (before language development) or postlingual (after language development) [23]. Hearing loss heredity can be classified as autosomal dominant (A), autosomal recessive (B), X-linked (X), or based on changes in the mitochondrial DNA. Cerebellar and vestibular disorders show not only overlapping clinical symptoms, but also shared genetic risk factors. Some types of autosomal dominant nonsyndromic deafness genes (DFNA) are known to be associated with vestibular symptoms (DFNA9, DFNA11 and DFNA15).

### Dfna9

DFNA9 is a nonsyndromic autosomal dominant SNHL with vestibular dysfunction caused by heterozygous mutations in the *COCH* (coagulation factor C homology) gene, encoding the secreted protein cochlin. The protein contains an N-terminal signal peptide (SP), an LCCL (limulus factor C, cochlin and late gestation lung protein Lgl1) domain, two von Willebrand factor A-like (vWFA) domains and two short intervening domains (ivd) [24]. It is an adult-onset form of progressive high-frequency SNHL associated with variable vestibular dysfunction that consisted of gait imbalance with instability in the dark and oscillopsia. Vestibular testing showed bilateral vestibular hypofunction [25].

Robertson et al. [26] identified a partial human cDNA for a novel cochlear transcript, hCoch-5B2, later called COCH. High levels of hCoch-5B expression were only seen in human fetal cochlea and vestibule, within a large panel of human fetal and adult tissues. They mapped hCoch-5B2 to human 14q11.2–q13 linked to DFNA9. Additionally, they reported three missense mutations in human *COCH*, in three unrelated kindreds with DFNA9. All three residues mutated in DFNA9 were conserved in mouse and chicken Coch and were found in a region containing four conserved cysteines with homology to the LCCL domain. Human temporal bones show histopathological findings of an acidophilic ground substance in DFNA9 patients [27].

So far, 21 mutations have been described in the *COCH* gene. Bae et al. [24] performed a comprehensive analysis of clinical information and molecular findings from DFNA9 patients to identify genotype–phenotype correlations. Five mutations were limited to intracellular cochlin: two vWFA domain mutants that formed high molecular weight aggregates in cell lysates, and three LCCL domain mutants, which were detected as intracellular dimeric cochlins, resulting in earlier age of onset of hearing defects. Further, those with vWFA domain mutations exhibited predominantly hearing loss, while LCCL domain mutations showed accompanying vestibular dysfunction. So, the failure of mutant cochlin transport, which impairs cochlin secretion, induces the formation and retention of dimers and large multimeric intracellular aggregates, which correlates with an earlier onset and progression of hearing loss in DFNA9.

Furthermore, Tsukada et al. [28] reported two novel mutations in p.I372T and p.C542R. The patients with the novel mutations in p.I372T and p.C542R within the vWFA2 domain showed early-onset progressive hearing loss, and those with the p.G88E mutation showed late-onset hearing loss and acute hearing deterioration over a short period. Vestibular symptoms were reported in the patients with p.G88E and p.C542R variants. Vestibular testing was carried out for the family with the p.G88E mutation. The patient had a severe vestibular dysfunction, while his son had no vestibular symptoms and a normal bilateral response in cVEMP, thought he showed a unilateral semicircular canal dysfunction with mild hearing loss.

### Dfna11

DFNA11 is a nonsyndromic form of progressive SNHL with postlingual onset caused by *MYO7A* gene (myosin VIIA, 11q13.5), a further gene known to be associated with vestibular dysfunction. *MYO7A* is an unconventional myosin involved in the structural organization of hair bundles at the apex of sensory hair cells [29]. DFNA11 is characterized, clinically, by low- and middle-frequency hearing loss and variable vestibular dysfunction [30]. Patients have a late-onset hearing loss, and it progresses to severe impairment. Several mutations in the *MYO7A* gene have been identified as a cause of DFNA11 [31], and whole genome-sequencing approaches seem to validate this association [32]. Tamagawa et al. [33] described a family with 19 members, eight of which had bilateral progressive hearing impairment. Genetic studies demonstrated that all eight subjects presented a deletion in *MYO7A* gene, confirming a complete penetrance in the family. The subjects presented bilateral SNHL at all frequencies, five of them showed spontaneous nystagmus while the other three showed bilateral caloric hyporeflexia.

### Dfna15

DFNA15 is characterized by early-onset progressive high-frequency hearing loss, but also by variable vestibular phenotype. It is caused by several missense mutations in *POU4F3 gene* (POU class 4 homeobox 3, 5q32). This gene encodes a member of the POU-domain family of transcription factors. POU-domain proteins have been observed to play important roles in control of cell identity in several systems [34]. Two affected individuals presented vestibular symptoms, according to their medical history. Vestibular examination (electronystagmography with rotatory chair and caloric tests) in 18 carriers and one phenocopy carrier in a Dutch family with DFNA15 with a L289F mutation in *POU4F3* gene showed a great variability in the vestibular function from normal response to complete areflexia, suggesting that additional genes should be involved in the vestibular phenotype [35].

### Dfna28

DFNA28 is a progressive dominant SNHL associated with a frameshift mutation of grainyhead-like 2 (*GRHL2*), but its etiology and mechanism remain unknown. The protein encoded by this gene is a transcription factor that can act as a homodimer or heterodimer with either *GRHL1* or *GRHL3*. In 2002, Peters et al. [36] first associated the DFNA28 locus with mild-to-moderate postlingual progressive bilateral SNHL involving an affected five-generation North American family. Affected members had a heterozygous c.1609_1610insC mutation in exon 13.

Han et al. [37] developed a zebrafish grhl2b (T086) mutant model in which grhl2b expression was interrupted by an insertion of a Tol2 transposon element. The mutants exhibit enlarged otocysts, smaller or absence of otoliths, malformed semicircular canals, insensitiveness to sound stimulation and, interestingly, imbalanced swimming motion. Since grainyhead-like family members can regulate epithelial adhesion, the expression of some genes encoding junction proteins in mutants was examined. They showed that the expression of claudin b (cldnb) and the epithelial cell adhesion molecule (epcam) was abolished or dramatically reduced, and apical junctional complexes were abnormal in otic epithelial cells of mutant embryos. Co-injection of cldnb and epcam mRNA could largely restore the mutant phenotype. Injection of human wild-type *GRHL2* mRNA could rescue the inner ear phenotype, but not the mutant *GRHL2* mRNA derived from DFNA28 patients, into grhl2b (T086) mutant embryos.

*GRHL2* has been confirmed as a deafness gene at the DFNA28 locus. Together with *GRHL1,* these two homologous proteins have similar sequences and functions. A grhl1 down-regulated zebrafish model exhibited inner ear developmental malformations, including missing otoliths, disordered and abnormal number of hair cells in the inner ear and lateral line, and sound insensitivity [38]. Remarkably, the mutant zebrafish swam in circles, being hair cell apoptosis evident. Using electron microscopy, desmosomes in the otic sensory epithelium were found to be damaged. These defects were partially rescued by treatment with either *GRHL1* or its target gene, DSG1 [38].

### Dfnb102/103

DFNB102/103 has been described in a Turkish family with recessive hearing loss and vestibular dysfunction. *CLIC5* (chloride intracellular channel 5, 6p12.3) gene encodes a member of the chloride intracellular channel (*CLIC*) family of chloride ion channels. The encoded protein associates with actin-based cytoskeletal structures and may play a role in multiple processes, including hair cell stereocilia formation, myoblast proliferation and glomerular podocyte and endothelial cell maintenance [39].

The mutation was identified in a consanguineous family diagnosed with autosomal recessive SNHL. A homozygous region of 47.4 Mb on chromosome 6p21.1–q15 was shared by the two affected siblings. This region contains 247 genes including the known deafness gene *MYO6,* but no pathogenic variants were found. Subsequent candidate gene evaluation revealed *CLIC5* as an excellent candidate gene. The orthologous mouse gene is mutated in the jitterbug mutant that exhibits progressive hearing impairment and vestibular dysfunction. Mutation analysis of *CLIC5* revealed a homozygous nonsense mutation c.96T > A [p.(Cys32Ter)] that segregated with hearing loss. Hearing impairment in the present family had an early childhood onset and progressed, from mild to severe, before the second decade. Impaired hearing was accompanied by vestibular areflexia and in one of the subjects with mild renal dysfunction [40].

### EVAS

Enlarged vestibular aqueduct syndrome (EVAS) is the most common form of inner ear malformation caused by a dilated vestibular aqueduct. It has been associated with a mutation in the *SLC26A4* gene, which encodes pendrin, an anion exchanger expressed in the endolymphatic sac, in different epithelial cells of the cochlea and vestibular labyrinth, as well as in the kidney and thyroid gland. Mutations in this gene cause DFNB4 and Pendred’s syndrome, both of which are related to EVAS [41]. Some studies, performed in Chinese population, analyzed *SLC26A4* gene using direct sequencing observing a total of 23 pathogenic mutations, 13 of which were unique [42], while other studies found a correlation between *SLC26A4* mutations with wider aqueducts at the midpoint and more severe hearing loss [43]. Nevertheless, recent studies [44, 45] did not show any correlation between *SLC26A4* gene mutations and the nature, degree, and progression of hearing loss [46]. *SLC26A4* gene therapy targeted to the endolymphatic sac restored hearing and balance in *SLC26A4* mutant mice [47].

### Ménière’s disease

Ménière’s disease (MD) is a clinical syndrome that affects the inner ear, and it is characterized by episodes of spontaneous vertigo, usually associated with unilateral fluctuating SNHL, tinnitus and aural fullness [48]. The prevalence of MD is about 0.5–1/1000 individuals, being most of patients with MD considered sporadic, although around 9–10 % are familial cases in European descendant population [10, 49, 50]. Both familial and sporadic cases are clinically indistinguishable [2, 10]. The disorder is associated with an accumulation of endolymph in the scala media of the cochlea and the saccule (endolymphatic hydrops), and it may involve both ears in 10–40 % of patients [51]. Several triggering factors such as allergens, virus or autoantigens have been proposed as contributors to the pathophysiology of the endolymphatic hydrops in susceptible individuals, but no genetic locus has been linked with sporadic MD.

Several comorbidities are associated with MD, including migraine, obesity, psoriasis and systemic autoimmune disorders [52]. Although its exact etiology and pathogenesis are still unknown, and 1/3 of MD cases may have an anomalous response of the innate or adaptive system, the mechanisms involved remain unknown [53]. This hypothesis is supported by some evidence, including the response to steroids therapy, the finding of elevated levels of autoantibodies or circulating immune complexes (CIC) in the serum of some patients with MD against inner ear antigens. Remarkably, several allelic variants in immune innate response genes *MICA*, *TLR10* and *NFKB1* influence the hearing outcome in sporadic MD [54–56].

The hypothesis of a channelopathy has supported aquaporins (AQP) as molecular targets in MD. These proteins are water channels, the function of which is to transport water and solutes along the osmotic gradient, and four of them (AQP 1–4) are expressed in the human inner ear [57]. Water permeability of AQP is regulated by vasopressin [58], which has been reported to be increased in MD subjects before a vertigo attack. Vertigo attacks may arise from interplay of increased vasopressin release and allelic variants in AQP genes [59]. To date, genotyping studies on AQP have produced controversial results in *AQP2* and *AQP4* genes [58], and a homozygous c.105G- > C synonymous variant in *AQP3* was found in 11 of 34 patients with MD [60].

Moreover, the association with allelic variants of two voltage-gated potassium channel genes, *KCNE1* and *KCNE3* in Japanese population, produced contrasting results, and it could not be replicated [61, 62].

Finally, in a case–control study, the predisposition to develop MD was linked to a common variant in the *ADD1* gene, encoding a cytoskeletal protein, which interacts with the Na^+^, K^+^, -ATPase transporter, leading to its faster activity [63]; however, these findings were not replicated in an independent cohort [64].

### Familial Ménière’s disease

Familial MD (FMD) should be considered if at least two family members (first or second degree) fulfill all the criteria of definite or probable MD [10]. Most families described have an autosomal dominant pattern of inheritance, but, as FMD shows clinical heterogeneity, mitochondrial and recessive inheritance patterns are also observed in some families [10]. Linkage studies in FMD have found candidate loci at 5q14–15 in a German family [65] and 12p12.3 in a large Swedish family [66], but the genes were not identified. By exome sequencing, Requena et al. [67] have identified mutations in *DTNA* and *FAM136A* genes in an autosomal dominant Spanish family with MD segregating the phenotype in three women in consecutive generations. *DTNA* encodes alpha-dystrobrevin, a dystrophin-associated protein which interacts with transmembrane proteins and actin in the basolateral membranes of epithelial cells, and it is associated with tight junction reorganization [68]. The identified mutation at position chr18: 32462094G > T in the *DTNA* gene is a rare splice-site acceptor skipping in exon 21. *FAM136A* is a mitochondrial protein of unknown function, and the mutation chr2:70527974C > T leads to a novel stop codon which shortens the protein product. Both *FAM136A* and *DTNA* proteins are expressed in the neurosensorial epithelium of the crista ampullaris of the rat. Further studies are needed to investigate *DTNA* and *FAM136A* genes in sporadic MD.

## Conclusion

The improvement in phenotyping families with vestibular symptoms combined with exome sequencing will facilitate the identification of rare variants and genes involved in familial vestibular disorders.
